# Screening for breast and cervical cancer among OST patients: a qualitative study of barriers and suggested interventions to increase participation

**DOI:** 10.1080/17482631.2023.2175767

**Published:** 2023-02-08

**Authors:** Lars Garpenhag, Disa Dahlman

**Affiliations:** a Center for Primary Health Care Research, Department of Clinical Sciences Malmö, Lund University/Region Skåne, Malmö, Sweden; b Faculty of Medicine, Department of Clinical Sciences Lund, Psychiatry, Lund University, Lund, Sweden

**Keywords:** Women, opioid substitution treatment, drug use, cancer screening, health equity, focus groups, Sweden

## Abstract

**Purpose:**

Women with current or previous drug use are at risk of poor breast and cervical cancer outcomes. While screening is known to decrease cancer mortality, screening participation is sparsely investigated among drug dependent women. The aim of this study was to explore experiences of breast and cervical cancer screening—including barriers and suggested interventions to promote increased participation—among women in opioid substitution treatment (OST).

**Methods:**

Three focus group interviews were conducted at one OST clinic in Malmö, Sweden. The interviews were moderated by OST staff, assisted by a researcher. A descriptive qualitative analysis was carried out using a template analysis approach, employing a model of healthcare access to organize the description of barriers.

**Results:**

The 11 participants reported several barriers to screening access, affecting the perceived need of screening and the opportunities to seek and reach screening services. Some barriers appear to be specific to women with previous or current drug use. Suggested interventions were moral and practical support, integrated/specialized delivery of screening services, and enhanced screening invitation procedures.

**Conclusions:**

The study findings provide insight to difficulties with screening compliance among women with current or previous drug use, and provide a knowledge base for quantitative and intervention studies.

## 1. Introduction

Female breast cancer has the highest incidence of all cancer forms, and cervical cancer has the fourth highest incidence in women worldwide (Sung et al., 2021). Early detection through screening has shown great potential to reduce the mortality rates of these cancers. In Sweden, screening for both breast and cervical cancer are subject to nationally mandated programs, and is offered free-of-charge. The National Board of Health and Welfare recommends regular screening at certain intervals to all female residents in ages 40–74 for breast cancer, and ages 23–64 for cervical cancer. The coverage of these screening programs has increased over time, and participation rates are high overall, albeit with considerable regional and local variation (Cancerfonden, 2021). Since the introduction of screening, there has been a significant drop in breast cancer mortality (Socialstyrelsen, 2014), while cervical cancer has decreased in both incidence and mortality (Socialstyrelsen, 2020).

Recent research suggests that the risks of developing breast and cervical cancer are not evenly distributed throughout the population, and studies show that Swedish women with current or previous drug use—here abbreviated as WWUD (women who use/have used drugs) – are particularly vulnerable. Compared to the general population, Swedish WWUD not only suffer an increased risk to develop and die from breast cancer (Dahlman, Magnusson, et al., 2021), but also display a greater incidence of cervical cancer (Dahlman, Li, et al., 2021). Internationally, studies have made similar findings regarding the increased vulnerability of women with different forms of substance use. Ahlgrén-Rimpiläinen and colleagues found an increased breast cancer mortality among Finnish women with substance use disorders (Ahlgrén-Rimpiläinen et al., 2020), and Australian studies have shown that WWUD have an increased risk of cervical cancer and cervix cytological abnormalities (Kricker et al., 2013; Reece, 2007; Soccio et al., 2015). One Australian study found great excess mortality from cancers in people with opioid dependence specifically, which included an elevated risk to die from cervical cancer (Randall et al., 2011). Importantly, various forms of drug use have been found to be associated with behaviours that may increase the risk to develop breast and cervical cancer, like smoking (Troberg et al., 2019) and sexual risk behaviours (Allen et al., 2020; Hwang et al., 2000).

The increased risks associated with breast and cervical cancer among WWUD indicate the crucial importance of screening attendance in this group. In Sweden, screening compliance and experiences of WWUD remain unknown, despite an abundance of studies on Swedish women in general (Norfjord Van Zyl et al., 2018; Norfjord van Zyl et al., 2020; Oscarsson et al., 2008; Sterlingova & Lundén, 2018; Östensson et al., 2015). Internationally, studies on cervical screening point towards lower rates among WWUD both in and outside of OST treatment (Abrams et al., 2012; Haddad et al., 2015), as well as a lower likelihood of a recent screening in women currently using illicit drugs (Kricker et al., 2013). These results fit well into a wider picture of high levels of unmet health needs, and lacking access to health care—including preventive care—among people who use drugs (Chitwood et al., 2002; Drumm et al., 2003; Heinzerling et al., 2006; Hyshka et al., 2017; Miller-Lloyd et al., 2020; Powelson et al., 2014; Spithoff et al., 2019; Troberg et al., 2019). Therefore, it is plausible to expect that low screening compliance and late healthcare seeking might be factors that at least partly explain the increased risks for breast and cervical cancer in this population.

In this pilot study, we examined experiences, attitudes and beliefs associated with screening for breast and cervical cancer in a specific sub-group of WWUD: women enrolled in opioid substitution treatment (OST). First, we explored the barriers that have negatively influenced OST patients’ participation in Pap smear and mammogram examinations—in the present as well as in past periods of active drug use—as reported by the women themselves, and organized them using an established model of healthcare access. Second, we map research participants’ suggestions on interventions that could serve to increase screening participation among WWUD. As this marks the first attempt to study this subject, the purpose was to generate insight that will guide the development of a survey instrument, as well as hypotheses for further research. The rationale behind the choice to study OST patients was that they have experiences of living with both non-treated and treated substance dependence. In addition, they form a sub-population of WWUD who are in regular contact with the healthcare system, which simplifies outreach with interventions.

## 2. Materials and methods

We used a qualitative thematic approach to analyse data collected through focus group interviews with female patients enrolled in OST. Prior to commencement the study protocol was reviewed and approved by the Swedish Ethics Board (file no. 2020–04150). All interviewees received oral and written information about the study, and provided informed, written consent of study participation.

### 2.1. Recruitment and data collection

Data was collected through focus groups interviews, a technique for data collection particularly suitable for exploratory studies (Peek & Fothergill, 2009). Recruitment was carried out at a clinic in Malmö, Sweden. Malmö is Sweden’s third largest city (c. 350 000 residents), and part of the transnational metropolitan Öresund region (c. 4 million residents). At the time of the study, the city had six OST clinics, serving approximately 540 patients. The clinic where the study was conducted is operated by a private provider, but offers tax-financed services on the behalf of Region Skåne (the county council of Skåne).

A criterion sampling approach was employed in the recruitment of participants (Moser & Korstjens, 2018). Inclusion criteria for participation were female sex, enrolment in OST at the clinic and a minimum age of 23 (the minimum age of the screening program for cervical cancer). Persons who did not speak Swedish, were notably intoxicated, or suffered from mental health issues that preclude meaningful participation (as judged by clinic staff) were excluded from the study. Using a key informant recruitment approach (Peek & Fothergill, 2009), prospective participants were invited to the study by clinic staff. Staff members also decided the group composition, with the aim to create dynamic groups without known conflicts between individual participants. Since the studied population forms a marginalized group, and the topic of discussion was potentially sensitive, the group size was intentionally kept smaller (3–5 participants per group) than what is often suggested as an ideal for focus group studies (e.g., 8–10) (Peek & Fothergill, 2009).

Three interviews were conducted, with a total of 11 female OST patients (out of a total of 42 enlisted at the clinic at the start of the study), aged 24–58 years (all were eligible to be called up for cervical cancer screening, four for breast cancer screening). Interviews were initiated in the fall of 2020, but had to be put on hold due to restrictions brought by the COVID-19 pandemic. One final interview was conducted in the fall of 2021.

The interviews were semi-structured, and based on a predetermined interview guide containing questions about participants’ experiences and views regarding screening for breast and cervical cancer, circumstances that had hindered access to such services, and thoughts about what could make it easier for them to adhere to guidelines for screening (please see supplementary file for the interview guide, translated to English by the first author). An OST counsellor moderated the interviews on-site at the clinic, while the first author managed recording equipment, kept notes and posed occasional follow-up questions. The interviews were audio recorded and transcribed verbatim, leaving out only names of individuals. Upon completion of the interviews, participants received a grocery store gift cards to a value of 200 SEK (appr. 25 USD).

### 2.2. Analysis

A template analysis (TA) approach (King, 2004) was employed to create a qualitative description (Sandelowski, 2000) of participants’ experiences and views. TA has been described as “a systematic technique for categorizing qualitative data in hierarchical clusters” (Brooks et al., 2013, p. 3), in which themes—categories created a priori or derived from the data—are organized in a coding template to lend structure to the analysis. After a comprehensive reading of all the data to create familiarity, preliminary coding was initiated. *Perceived barriers to screening access*, and *suggested interventions to facilitate participation* were used as a priori categories, providing an initial structure for the analysis. In order to make sense of how specific barriers affect access in different ways, codes related to barriers were clustered in accordance with the five dimensions of healthcare access identified by Levesque, Harris and Russell (Levesque et al., 2013). The analysis was thus based on a notion of access to healthcare as processual—beginning with the prospective patient desiring care and ending with the consequences of the health care encounter—and as shaped at each stage by an interaction between abilities of the patient and factors deciding the accessibility of healthcare services (Table 1). Themes derived from the data were then incorporated within this framework. An initial coding template was produced based on a subset of data and then refined through it being reiteratively applied to the whole dataset, before it was finalized. Initial coding and creation of a template were carried out the first author, while the second author reviewed both the framework and the analysis before it was finalized (the final template can be obtained by request). To protect the identity of the participants who are quoted, we refer to them using fictitious names.

**Table I.** A processual and interactive model of healthcare access.

Stages in the process of access	Characteristics that shape access	
*The individual*	*Patient characteristics*	*Service characteristics*
…perceives need	Ability to percieve need	Approachability
…seeks care	Ability to seek care	Acceptability
…reaches care	Ability to reach care	Availability and accomodation
…utilizes care	Ability to pay	Affordability
…is offered services	Ability to engage	Appropriateness
Based on (Levesque et al., 2013)		

## 3. Results

All but one of the participants had experienced either a Pap smear or a mammogram, and four of them had done both. In no case did their screening histories adhere to the officially recommended intervals. For some, the deviations from the recommended intervals were considerable. For instance, one participant in her fifties had undergone her first mammogram mere weeks before the interview, and had not had a Pap smear taken since the 1990s.

Most participants reported feeling confident in their knowledge of the general functions and benefits of mammograms and Pap smears, and those who expressed any principled opinion about the screening programs all agreed on their importance. Feelings associated with the screening procedures in themselves, however, diverged considerably, ranging from largely positive to very negative. Those who were content identified quick and impersonal procedures and a friendly reception as factors that had helped shape the experience into a positive one. Those with negative experiences cited circumstances like painful or rough examination techniques, a lack of information about what was going to happen, stressful procedures, a mechanical atmosphere and general discomfort as reasons for their discontent.

### 3.1. Perceived barriers to screening access

Participants presented a host of factors that had hindered them from participation in screening, all mainly affecting the first three stages of healthcare access, that is, the perception of need, the seeking of services and the reaching (timely attending) of services.

#### 3.1.1. Barriers to perceiving need of screening

A number of the identified barriers had in common that they had influenced participants’ assessment of the relative importance of getting screened, that is, the perceived need. Several participants reported a fear of cancer as a reason for why they had missed out on screening opportunities. More specifically it was a matter of not wanting to know one’s cancer status. For the same reason, one participant had been reluctant to undergo genetic testing to establish her cancer risks. If she was a high-risk individual, she argued, she simply did not want to know about it.

*Klara, in her thirties:*
I might have skipped out on it more out of fear. [—] Well, I’ve just thought that “no, now I have cancer—no, I don’t want to know.” So, I’ve built up something around it. So, that’s a large part of it.

*Märta, in her forties:*
That! I agree, I just have to tell you, I have cancelled that appointment six times!

Others had weighed the benefits of screening against other interests, and found it not to be a priority. To some, self-esteem issues had coloured such judgements, making them feel unworthy of the kind of self-care that screening represented for them. While it was fine to care for others, it was not as easy to expend the same amount of care to oneself, and thus the perceived benefits of screening participation were diminished.

You feel used up [as a PWUD]. [—] You don’t really feel worthy … or how to put it. You don’t have much self-esteem. And somehow you almost feel that it would be better if [the opportunity to be screened] goes to someone who … lives a proper life and is worth it.

*Britta, in her fifties*

In some cases, women had simply found other matters more pressing, not least during periods of active drug use. For several participants, the health risks presented by cancer had not appeared that significant put next to more immediate practical needs (e.g., to find accommodation) or health risks (e.g., to suffer an overdose).

I believe that it’s difficult when you’re so deep into your addiction that you … like, there are overdoses all the time, so, why would you care about perhaps dying from a disease when you’re close to dying from the drugs all the time, you know?

*Lena, in her twenties*

Not least, quite a few participants had experienced that activities directly tied to drug use had out-competed the need for preventive healthcare. First and foremost, this included the time-consuming hunt for drugs to maintain one’s addiction. However, intoxication in itself was cited as a barrier as well, due to direct negative effects on motivation, and for some also a reluctance to show up for appointments intoxicated.

*Klara, in her thirties:*
Well [as an active user] the only thing that matters is when to get your next fix.

*Sofia, in her thirties:*
You live in your own little world.

*Moderator:*
Yes, you’re in your own bubble?

*Klara, in her thirties:*
Yes.

*Märta, in her forties:*
[To attend screening] comes last in the list of priorities.

While several of these factors are more directly associated with active illicit drug use than with life as a compliant OST patient, a few of the participants pointed towards difficulties to draw a clear line between the two. One woman remarked that OST should not be considered an instant fix to all the problems you experience as an active drug user, and that even with such support it takes time to pick up the pieces of one’s life. Another felt that the priorities one makes as an active user tend to stick and become hard-wired, so that you continue to follow the same patterns.

#### 3.1.2. Barriers to seeking screening services

Other barriers had made screening services seem less attractive or acceptable due to attitudes and feelings held by the women themselves, as well as to their perceived relationship to health services. One factor that was cited as a deterrent was anticipated stigma. Participants felt distrust towards healthcare services in general, and envisioned visits to the hospital as threatening situations in which they risked to stand out from other patients and become subject to prejudice or even ill treatment because of their current or previous drug use. Because of the close associations with injection drug use, several participants were certain that having a history of blood borne diseases negatively influenced the attitudes they met from healthcare staff as well.

I have missed appointments … or made a conscious choice, not just because I’ve buried my head in the sand out of fear, but also because … I have been so damn poorly received by healthcare services […]. So, well I just assume that I will be ill-treated in the future as well.

*Märta, in her forties*

There is an inherent, ehm, fear, shame, I don’t know, that’s just there when you do the test at the hospital. Even if you haven’t used [drugs] in a long time, the way you are treated is still there, all the times you have been forced to wait in some bloody chair, and wait, wait, wait, while everyone else just breeze by.

*Ingrid, in her fifties*

While many participants had experienced both mammograms and Pap smears as uncomfortable procedures, this was not necessarily described as a barrier to future participation. However, some felt that the anticipated discomfort associated with undergoing an intimate and invasive examination was a major issue that made participation difficult. Previous traumatic experiences, in- or outside healthcare settings, were described as a circumstance that could contribute to such feelings.

Well, I have not [participated in screening], because I have my traumas deep down, and well, I wouldn’t have been able to take it. It makes me feel a bit like a failure to be honest, but I don’t know. [—] That I would go there and give up all control, when I’ve had like 99% poor interactions with doctors throughout the years. No, it is just so scary.

*Jonna, in her twenties*

#### 3.1.3. Barriers to reaching services

Yet another set of barriers acted through making it more difficult to reach and attend screening services. In this context, participants found that the unstable life associated with active drug use had created problems for them in the past. Transient living conditions, without a steady residence, made it difficult to even receive invitations to screening appointments. In addition, several women explicitly named the ability to maintain basic hygiene as a precondition for attending such examinations.

Such practical difficulties aside, several referenced their inability to handle their personal affairs as another complicating factor. Some would fail to regularly open their mail, and would even discard letters instantly if they suspected them to contain bills or official correspondence. Others cited difficulties in keeping track of appointment times as a problem.

[If you suffer from] addiction, you often live an unstable life. Perhaps you don’t have an address or anything. You’re … You don’t live like you’re supposed to do in a society, opening your mail or you know … no, you live to survive the day. You’re not part of normal life.

*Britta, in her fifties*

While not figuring as prominently in participants’ discussions, difficulties associated with comprehending health information, and navigating the healthcare system, were mentioned as well. For instance, one participant found it hard to understand the contents of invitations to healthcare appointments, as well as medical terminology in general. Another participant had been left bewildered and frustrated after several failed attempts to secure a screening appointment.

Now, when I’ve finally gathered myself and decided to do it, I’ve tried to call and book an appointment. But I’ve just been … I don’t know where I’m supposed turn for the booking, because I’ve called this place and they’ve just told me that, “no, this is wrong, you should call this other place”, and I’ve gone on to call them. And even when I’ve had [help from an OST assistant nurse], it has been … it is so complicated.

*Märta, in her forties*

### 3.2. Suggested interventions to facilitate screening participation

Participants suggested several kinds of interventions that they believed would make it easier for them to attend Pap smear and mammogram examinations. Their suggestions concerned three main types of measures: moral and practical support targeting women in addiction treatment, integrated or specialized service delivery, and enhanced invitational procedures.

#### 3.2.1. Moral and practical support

Participants requested two types of support to be routinely provided to patients enrolled in OST or other addiction care: moral and practical. In fact, several participants had already received such support from addiction services staff, but on an *ad hoc* basis, and thus, such help was not openly available to everyone on equitable terms.

First, they would like to see organized moral or psychological support, aimed at making WWUD feel more confident and motivated to participate in screening. Concretely, what was discussed was the need to have someone there to encourage and help them to prepare mentally beforehand, follow and keep them company during the appointment, and be there for them afterwards, during the wait for the results. Several participants emphasized the importance of having such support during the whole screening process, rather than just at the time of the appointment.

Secondly, several participants called for support in practical matters, e.g., help to keep track of appointment times, or provision of know-how and assistance in contacts with healthcare services. While most of the discussions about support centred on what treatment staff could do, the idea to organize peer groups for similar purposes was raised as well.

It would have been good if … some treatment facility, like, [the OST clinic] for instance, that you could ask us about it, keep check on it, and give support if needed, in regular intervals. There is [a named OST staffer] that I have felt so much support from, and I don’t think that I would have been there yet if it hadn’t been for her. So maybe, a little push from you is all we need. [—] And then, that someone can accompany you too, if it is really difficult.

*Märta, in her forties*

*Moderator:*
So, support to get there …

*Susanna, in her thirties:*
Yes.

*Moderator:*
… and to prepare …

*Susanna, in her thirties:*
Mhm.

*Moderator:*
… and, mhm, perhaps in the contact in itself.

*Ingrid, in her fifties:*
Yes, and if you have to await a result, the fact that you have gotten through all this that you found to be so tough doesn’t mean it’s over, since you might need support and help in case the result doesn’t arrive as promised. Then you can help out, make calls, so that one doesn’t have to do it on one’s own. So, you should be accompanied through the whole event, until the whole thing is over.

#### 3.2.2. Integrated or specialized delivery of screening services

Several participants already had positive experiences of both integrated and specialized delivery of physical healthcare. Some of them had been in contact with the Ambulatorium, a unit for women’s healthcare providing maternal care to WWUD in collaboration with social services. Others were enrolled in PRIO, a local project for the delivery of on-site primary care at OST-clinics. It was suggested that similar solutions could help increase screening participation, by making it possible to avoid the stigma associated with visits to the hospital. One proposal was that a special women’s clinic for WWUD should be created, where women could both escape the stigma and meet healthcare staff who were used to having them as patients. Another proposed solution to the problem with stigma in healthcare was to offer screening services on-site at addiction care units.

I believe that if you’re actively using, to then go to a place, you know, if you look like you do … Perhaps you haven’t had a shower for three days, or you’re looking like totally emaciated or you’re under the influence, then you don’t want to go to the hospital and just stand around … But on the other hand, to visit a kind of place that offers another environment … maybe you can’t have places like that everywhere, but you actually could, since drug addiction is such a big … common illness.

*Lena, in her twenties*

I like to visit the Ambulatorium. I’m not sure if it’s possible only when you’re pregnant or if you would be eligible just by being a drug user, or ex-drug user? It is great to do tests there, because you don’t have to feel … or me myself, I don’t feel like … They work with drug users and ex-drug users, so you don’t have to feel ashamed or anything, and those who work there—like [the midwife] whom I’m sure many of you know … I feel, it is good for us who are in OST, the Ambulatorium is great.

*Elsa, in her thirties*

#### 3.2.3. Enhanced procedures for invitations to screening

Finally, participants had ideas about how the invitations to the national screening programs could be amended to make them more accessible to WWUD. One suggestion was to redesign the invitation letters to make them stand out, for instance through using envelopes in a stark colour, so that you would not mistake them for unwanted mail. Another was to make more efficient use of digital means of communication, based on the rationale that WWUD would be more easily reached through email or text messages, than by regular mail.

Well, yes you get [invitation] in the mail, but I believe that they could modernize things, you know, so that you receive them some other way. Not everyone has a steady address, and the postal services actually do mistakes, so that you miss appointments. [—] Not everyone has a digital mailbox either, but they should be able to modernize it in some way. At least they could send reminders.

*Lena, in her twenties*.

*Ingrid, in her fifties:*
They shouldn’t use a white envelope that …

*Moderator:*
No. What should the information look like?

*Ingrid, in her fifties:*
Well, it should be a pink envelope.

*Moderator:*
Yes, well … <laughs>

*Ingrid, in her fifties:*
Yes … so that you can know, this is not from the Enforcement Authority […].

*Klara, in her thirties:*
No, exactly.

*Ingrid, in her fifties:*
No, this is for *me*.

## 4. Discussion

In this study, a wide range of perceived barriers to breast and cervical cancer screening among Swedish WWUD have been identified. These findings are novel, since, to the best of our knowledge, it is the first study to explore WWUDs experiences of barriers to these forms of cancer screening services. A number of factors, some of them tightly associated with living as a WWUD, had affected participants’ perceptions of their need for screening. A fear of knowing one’s cancer status, as well as feelings of unworthiness, had made screening attendance less of a priority. So did the primacy of maintaining a drug dependence, and the precariousness of daily WWUD life. Meanwhile barriers decreasing the acceptability of screening services, in the form of anticipated stigma and discomfort, were cited as reasons to refrain as well. Finally, unstable life style and living conditions, having a poor grasp of one’s personal affairs, and having trouble comprehending healthcare and health information, were all described as barriers, mainly affecting the practical possibilities to go through with a screening appointment. Thus, we found barriers that affect the first three stages in the process of accessing healthcare, influencing the perception of needs for screening as well as the opportunities to seek and actually be able to reach and utilize such services. Meanwhile, neither costs nor the appropriateness and quality of services, were brought up.

Some of the factors appear to be specific to WWUD. As noted by Drumm and colleagues (Drumm et al., 2003) in a study on illicit drug users, a life-style centred on the acquisition and consumption of drugs tends to make other needs—like the need for healthcare—pale in comparison. Our findings suggest that, beside the effect of this so-called “master role of the addict” (Drumm et al., 2003, p. 468), the precariousness of WWUD life in general affects priorities. When immediate and apparent risks to your health become perceived as a part of everyday life, they easily trump any more forward-looking need for preventive healthcare. The anticipation of drug related stigma in healthcare encounters, and the perceived role of transient and unstable living conditions, may also be understood as barriers particular to the population under study. Still, similarities to previous findings regarding Swedish women in general can be noted as well. For instance, the tendency to disregard one’s own interests and needs to the benefit of those of others has been reported as a factor that may affect breast cancer screening behaviour (Norfjord van Zyl et al., 2020). Neither is it unique to WWUD to harbour negative feelings about screening procedures, or have interests or responsibilities that may conflict with the need to utilize preventive care (Oscarsson et al., 2008; Sterlingova & Lundén, 2018).

Some types of perceived barriers that have been observed among other women, meanwhile, are conspicuously absent in our findings. For instance, while screenings for breast and cervical cancer are free of charge in Sweden, other costs, e.g., tied to transportation have been shown to be a potential issue (Norfjord Van Zyl et al., 2018). One plausible way to interpret this discrepancy in light of our findings is that the processual access to screening among WWUD tend to be cut short at an earlier stage, removing much of the relevance of such problems. However, it is also notable that the study was conducted in an urban area with easy geographical access to service providers.

Study participants allowed welcome insight to what they themselves found to be acceptable solutions to the problems they described. They suggested interventions in the form of routines for moral and practical support to facilitate screening, specialized delivery and enhanced invitational procedures for the national screening programs. These interventions took aim at mitigating pragmatic problems, but also at making it emotionally easier to participate in screening, and to lessen the risk of stigma that participants associated with the utilization of healthcare services in general.

In the design of an intervention to facilitate access to a healthcare service, it is important to consider how barriers can be more or less amenable to change. Levesque, Harris and Russell (Levesque et al., 2013) argue that although both individual and service characteristics shape access, feasible short-term solutions to lacking access do best at transforming the latter. Similar conclusions have been drawn by Andersen (Andersen, 1995) in regard to the implications of the seminal Behavioural model of health services use. Considering the mutability of different factors that influence healthcare access, enabling factors—such as the allocation of resources that makes utilization possible—form a more malleable variable than both demographic structures and patients’ health beliefs (Andersen, 1995). In this sense, the suggestions made by our study participants are reasonable and worth consideration. However, while this study has given insight into a range of experiences concerning barriers to screening among WWUD, our explorative analysis says nothing about the relative significance of different barriers. Thus, more studies are needed to see where and how resources can be put to use in an efficient manner. The findings of this study thus imply a need for larger-scale, quantitative studies addressing screening compliance, barriers, and facilitators in women in OST and other WWUD.

### 4.1. Strengths and limitations

The major strength of this study is that it has successfully collected data from a vulnerable and under-studied population, which can be used to understand inequities in screening service access and cancer incidence and survival. One limitation of the study is the small sample size, a result of planning and recruitment being hampered by the ongoing pandemic. While it cannot be ruled out that a larger sample could have rendered deeper insights into the experience of WWUD, we believe that the collected data is rich and varied enough for the explorative aims and scope of our study.

5.

## 5. Conclusion

Women in OST report a host of factors that constitute barriers to cancer screening, mainly affecting the perceived need of screening, and hindering them from seeking and successfully reaching screening services. Three explicit suggestions for interventions to minimize screening barriers were suggested by the participants: moral and practical support, integrated or specialized delivery of screening services, and enhanced procedures for screening invitation. The findings are novel and provide insight to WWUDs’ difficulties with screening compliance, but need to be evaluated in larger, quantitative studies.

## Supplementary Material

Supplemental Material
