# Supplementary material for: Screening for breast and cervical cancer among OST patients: a qualitative study of barriers and suggested interventions to increase participation
Source: Int J Qual Stud Health Well-being. 2023 Feb 8;18(1):2175767. doi: 10.1080/17482631.2023.2175767 (PMC9930823; doi:10.1080/17482631.2023.2175767)
Supplement: Supplemental Material [file ZQHW_A_2175767_SM1900.docx]

Interview guide for focus groups interviews on breast and cervical cancer screening

1. What is your first name, and how old are you?
2. a) Have you ever attended screening for breast cancer (a mammogram)?
   b) Have you ever attended screening for cervical cancer (a Pap smear)?
3. When was the last time you were screened for breast or cervical cancer?
4. Do you feel confident that you know what a mammogram/Pap smear is and what it is used for?
5. Those of you who have attended screening, what was your experience of the appointment?
6. Have you ever missed a screening appointment that you have been invited to?
7. Those of you who have missed screening appointments, why did you miss it?
   The whole group: Can you imagine other reasons that could make someone fail to appear for a screening appointment?
8. Those of you who have missed screening appointments, do you have ideas about anything that could have made it easier for you to actually attend screening?
   The whole group: Can you imagine other things that could make it easier to keep a screening appointment?
